# Machine Learning Prediction of Visual Outcome after Surgical Decompression of Sellar Region Tumors

**DOI:** 10.3390/jpm12020152

**Published:** 2022-01-25

**Authors:** Nidan Qiao, Yichen Ma, Xiaochen Chen, Zhao Ye, Hongying Ye, Zhaoyun Zhang, Yongfei Wang, Zhaozeng Lu, Zhiliang Wang, Yiqin Xiao, Yao Zhao

**Affiliations:** 1 Department of Neurosurgery, Huashan Hospital, Shanghai 200040, China; norikaisa@gmail.com (N.Q.); yezhaozj663812@126.com (Z.Y.); eamns@hotmail.com (Y.W.); 2 Neurosurgical Institute, Fudan University, Shanghai 200040, China; 3 National Center for Neurological Disorders, 985 Jinguang Road, Shanghai 201107, China; 4 Fudan University Graduate School, Fudan University, Shanghai 200043, China; yichenma@126.com; 5 Surgical Theatre, Huashan Hospital Hongqiao Campus, Shanghai 201107, China; wonderful1211@163.com; 6 Shanghai Clinical Medical Center of Neurosurgery, Shanghai 200040, China; 7 Shanghai Key Laboratory of Medical Brain Function and Restoration and Neural Regeneration, Fudan University, Shanghai 200040, China; 8 Department of Endocrinology, Huashan Hospital, Shanghai 200040, China; janeyhy@163.com (H.Y.); zhaoyunzhang@fudan.edu.cn (Z.Z.); 9 Department of Ophthalmology, Huashan Hospital, 12 Wulumuqi Zhong Road, Shanghai 200040, China; zzlu@fudan.edu.cn (Z.L.); zhlwang@fudan.edu.cn (Z.W.)

**Keywords:** pituitary adenoma, craniopharyngioma, optic chiasm, multicenter

## Abstract

Introduction: This study aims to develop a machine learning-based model integrating clinical and ophthalmic features to predict visual outcomes after transsphenoidal resection of sellar region tumors. Methods: Adult patients with optic chiasm compression by a sellar region tumor were examined to develop a model, and an independent retrospective cohort and a prospective cohort were used to validate our model. Predictors included demographic information, and ophthalmic and laboratory test results. We defined “recovery” as more than 5% for a *p*-value in mean deviation compared with the general population in the follow-up. Seven machine learning classifiers were employed, and the best-performing algorithm was selected. A decision curve analysis was used to assess the clinical usefulness of our model by estimating net benefit. We developed a nomogram based on essential features ranked by the SHAP score. Results: We included 159 patients (57.2% male), and the mean age was 42.3 years old. Among them, 96 patients were craniopharyngiomas and 63 patients were pituitary adenomas. Larger tumors (3.3 cm vs. 2.8 cm in tumor height) and craniopharyngiomas (73.6%) were associated with a worse prognosis (*p* < 0.001). Eyes with better outcomes were those with better visual field and thicker ganglion cell layer before operation. The ensemble model yielded the highest AUC of 0.911 [95% CI, 0.885–0.938], and the corresponding accuracy was 84.3%, with 0.863 in sensitivity and 0.820 in specificity. The model yielded AUCs of 0.861 and 0.843 in the two validation cohorts. Our model provided greater net benefit than the competing extremes of intervening in all or no patients in the decision curve analysis. A model explanation using SHAP score demonstrated that visual field, ganglion cell layer, tumor height, total thyroxine, and diagnosis were the most important features in predicting visual outcome. Conclusion: SHAP score can be a valuable resource for healthcare professionals in identifying patients with a higher risk of persistent visual deficit. The large-scale and prospective application of the proposed model would strengthen its clinical utility and universal applicability in practice.

## 1. Introduction

Pituitary adenomas (PAs) and craniopharyngiomas (CPs) are the most common brain tumors in the sellar region [1,2]. Patients complain of blurred vision when the tumor grows beyond the sella and compresses the optic chiasm. Optic nerve decompression by surgical removal of the lesion may result in visual function normalization in some patients but not in others [3,4,5,6].

The risks associated with persistent visual dysfunction include severe visual field defects, thin retinal nerve fiber layers, and pituitary macroadenomas. Careful evaluation of these risks plays a fundamental role in the clinical management of these patients. The identification of patients at high risk for persistent visual loss may be helpful as patients could be referred to further visual rehabilitation [7,8] as soon as possible after surgery. Moreover, it might serve as a cost-effective and straightforward means for preoperative patient–doctor communication.

Small sample sizes, unquantified outcomes, and partial predictors constitute the limitations of previous attempts to search for risk factors that predict for visual recovery after surgery [9,10,11,12,13,14,15,16,17,18,19]. However, the overall accuracy of these scores, along with their generalizability to external cohorts, remains modest, representing an unmet need for individualized patient management strategies.

From a clinical standpoint, the poor performance of existing risk scores might be related to insufficient predictive factors. Machine learning methods might overcome some of the limitations of current analytical approaches to risk prediction by applying computer algorithms to large datasets with numerous, multidimensional variables, capturing high-dimensional, non-linear relationships among clinical features to make data-driven outcome predictions. The effectiveness of this approach has been shown in several applications of sellar region tumors, where machine learning was superior in validating traditional risk stratification tools, including prediction endocrine remission after surgical or radio surgical treatment of acromegaly [20,21]. Thus, we sought to develop a machine learning-based model (Prediction of Visual Outcome in Sellar Tumors, PREVOST) integrating clinical and ophthalmic features to predict visual outcomes after transsphenoidal resection of sellar region tumors.

## 2. Methods

### 2.1. Data Sources

**Figure 1:**
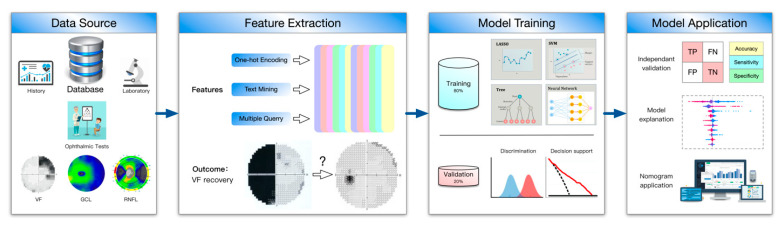
Overall study design.

To develop our machine learning models, we used a derivation cohort of 159 adult patients (≥18 years) with optic chiasm compression by a sellar region tumor with at least one year of follow-up. All of the patients suffered a visual field defect before surgery and were treated by transsphenoidal tumor resection and optic decompression in the Gold Pituitary Joint Unit (GPJU) between January 2019 to January 2021. The GPJU is a newly established unit that started in 2019 where patients with sellar region tumors are co-managed by a multidisciplinary team, including neurosurgeons, endocrinologists, and ophthalmologists. We excluded patients who were subtotally resected or patients who suffered a post-operation hemorrhage and needed an early emergent surgery. To test the generatability of our model, we used another retrospective cohort from Neurosurgical Institute of Fudan University (FNI), where surgeries and ophthalmic assessments were performed by different groups, to independently validate our model. We further validated our model in a prospective cohort admitted to GPJU from January 2021 to June 2021. Informed consent was obtained from patients at the time the data were collected. Predictors were assessed before surgery, and the outcome was assessed at follow-up. Institutional Review Board from both centers provided ethical approval. The overall study design is depicted in Figure 1.

### 2.2. Ophthalmic Examinations

Patients underwent a thorough ophthalmic examination by experienced ophthalmologists, including pupil, anterior, and posterior segment examination. Patients with other ocular diseases were excluded. Static automated perimetry was performed using the Humphrey 750 Visual Field Analyzer (Zeiss-Humphrey Systems, Dublin, CA, USA) and a central 30-2 threshold protocol. Fixation loss less than 20%, false-positive error less than 20%, and false-negative error less than 20% were ensured for a validated visual field. We documented the mean deviation (MD), pattern standard deviation (PSD), visual field index (VFI) on the report. The retinal nerve fiber layer (RNFL) thickness and ganglion cell layer (GCL) thickness were assessed by RTVue (Optovue, Fremont, CA, USA) using three-dimensional disc and optic nerve head (ONH) protocols.

### 2.3. Predictor Variables

Predictors were included based on a balance of clinical knowledge, past research, and likely clinical usefulness. The baseline model comprised visual acuity, MD (decibel, db), PSD (db), VFI (%), RNFL (μm), and GCL (μm). The full model comprised age (years), gender (female or male), BMI (kg/ m²), hypertension (yes or no), diabetes mellitus (yes or no), tumor height on MRI (cm), diagnosis (pituitary adenoma or craniopharyngioma), hemoglobin (g/L), red blood cell (1012/L), white blood cell (109/L), sodium (mmol/L), albumin (g/L), creatinine (μmol/L), ACTH (pg/mL), cortisol (μg/dL), prolactin (ng/mL), free thyroxine (pmol/L), and total thyroxine (nmol/L).

### 2.4. Outcome

Ophthalmic recovery after surgical decompression was categorized as a binary outcome according to the 3 to 6 month follow-up (static automated perimetry). Mean deviation in the follow-up visual field was compared with data from the general population (built-in data in the Humphrey 750 Visual Field Analyzer), and a *p*-value was calculated automatically. If the *p*-value was more than 0.05, we defined the outcome as “recovery”; otherwise, we defined the outcome as “not recovery”.

### 2.5. Model Training

We used multiple imputations using chained equations for missing data. Seven machine learning classifiers—linear absolute shrinkage and selection operator, support vector machine, linear discriminant analysis, random forest, gradient boosting, neural network, and ensemble model—were employed to generate seven models for the prediction. The internal performance was assessed by fivefold cross-validation, by which the dataset was randomly divided into five even groups and evaluation was performed on one group at a time using the model built on the remaining 80% of the data. Model performance was assessed by the mean area under the receiver operating characteristic curve (AUC), and the best-performing algorithm was selected. The final algorithm was validated on the two validation cohorts.

### 2.6. Calibration

The calibration of the model was assessed graphically with calibration plots. We also recorded the Brier score, an overall measure of algorithm calibration (scores > 0.25 generally indicating a poor model).

### 2.7. Decision Curve Analysis

A decision curve analysis was used to assess the clinical usefulness of our model by estimating net benefit [22]. The net benefit is a metric of true positives minus false positives at a given risk threshold. The risk threshold is the amount of tolerable risk before an intervention is deemed necessary (0.5 in our case). In clinical practice, patients at high risk of not recovering were likely refered to visual rehabilitation as soon as possible after surgery. We drew a decision curve plot to visualize the net benefit of our model over varying risk thresholds compared with intervening in all patients or intervening in no patients. Classical decision theory proposes that the choice with the greatest net benefit at a chosen risk threshold should be preferred.

### 2.8. Feature Importance

To determine the major predictors of outcome, the importance of each feature was measured from the final model. We used the SHAP (Shapley additive explanations) score, a game-theoretic approach to explain the output of any machine learning model [23]. It measures features contributing to pushing the model output from the base value (the average model output over the training dataset we passed) to the model output.

### 2.9. Visual Representation

We developed a nomogram, which allows for an interactive exploration of the effect of risk factors and their combinations on the visual outcome according to their PREVOST score. The choice of variables for nomograms was based on essential features ranked by the SHAP score.

### 2.10. Statistical Analysis

Continuous variables with normal distribution were described as mean and standard deviation. Continuous variables with non-normal distribution were described as a median and a range. Categorical variables were described as counts and proportions. We used the linear mixed-effect models for the comparison with the control to account for intra-eye correlation. All statistical analyses were completed with R software version 3.4.2 (R Foundation for Statistical Computing, Vienna, Austria).

## 3. Results

**Figure 2:**
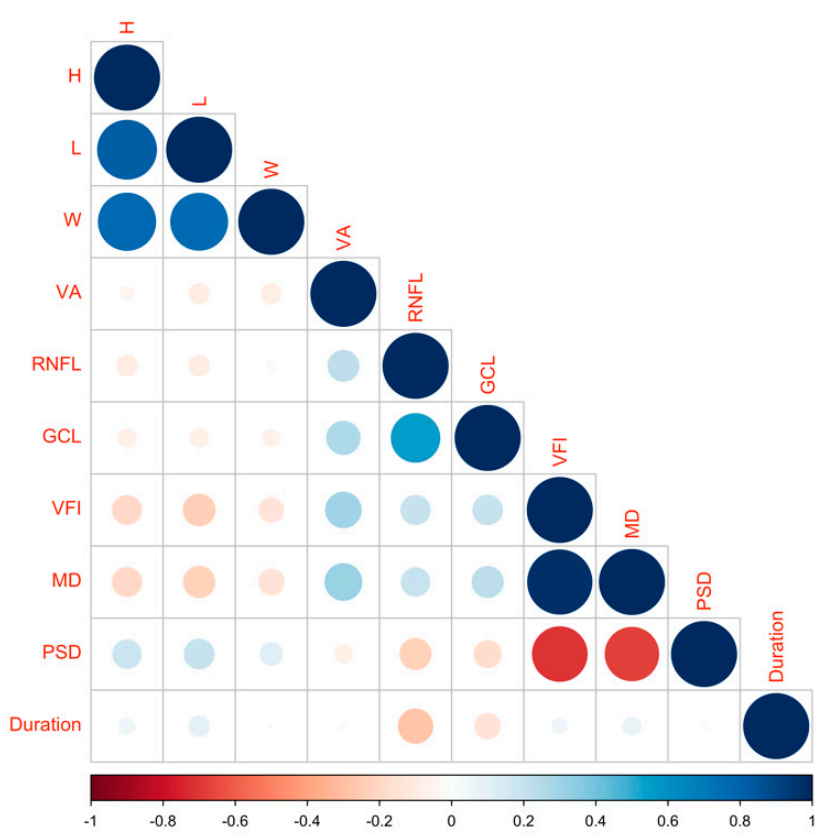
The correlation between visual severity, duration of symptoms, and size of the tumor. H: tumor height; L: tumor length; W: tumor width; VA: visual acuity; GCL: ganglion cell layer; VFI: visual field index; MD: mean deviation; PSD: pattern standard deviation.

The training cohort included 159 patients (91 male, 57.2%, Table 1). The mean age was 42.3 years old, and tumor volume was 9.4 (5.0–15.3) cm^3^. We included 96 patients with craniopharyngioma and 63 patients with pituitary adenoma in the analysis. Among the patients with pituitary adenoma, their pathologies [24] consisted of 33 gonadotroph adenomas, 13 corticotroph adenomas, 8 somatotroph adenomas, 6 lactotroph adenomas, 2 null cell adenomas, and 1 plurihormonal PIT-1 positive adenoma. High-risk adenomas included 13 silent corticotroph adenomas, 4 lactotroph adenomas in men, 3 sparsely granulated somatotroph adenomas, and 1 plurihormonal PIT-1-positive adenoma. In total, 318 eyes were included, 172 (54.1%) eyes out of 318 eyes recovered during early follow-up. The median change in mean deviation after surgery was 40.6% compared with pre-operation. Larger tumors (3.3 cm vs. 2.8 cm in tumor height, *p* < 0.001) were associated with worse prognosis than smaller tumors, and 73.6% of the eyes unrecovered were from patients with craniopharyngiomas compared with only 26.4% of the eyes unrecovered being from patients with PAs (*p* < 0.001). The laboratory test results were similar between recovered and unrecovered eyes. Eyes with better outcomes were those with shorter disease duration (6.0 months vs. 12.0 months, *p* = 0.002), better MD (−5.0 db vs. −14.6 db, *p* < 0.001), better PSD (4.3 db vs. 11.2 db, *p* < 0.001), and thicker GCL (60.5 μm vs. 56.6 μm, *p* < 0.001) before operation. Figure 2 shows the correlation between visual severity, duration of symptoms, and size of the tumor.

Furthermore, we looked at the difference between craniopharyngiomas and pituitary adenomas (Table 2). For the ophthalmological tests, the baseline mean deviation was −8.8 [−17.2–−4.0] db in the left eye and −7.8 [−15.9–−3.3] db in the right eye. Overall, though baseline ophthalmic examinations were similar for patients with CPs and PAs, PAs were associated with better prognoses.

Among all of the algorithms trained (Table 3), the ensemble model integrating all algorithms yielded the highest AUC: 0.911 [95%CI, 0.885–0.938]. The corresponding accuracy was 84.3%, with 0.863 in sensitivity and 0.820 in specificity. The random forest model and gradient boost model ranked second and third best regarding model performance.

**Figure 3:**
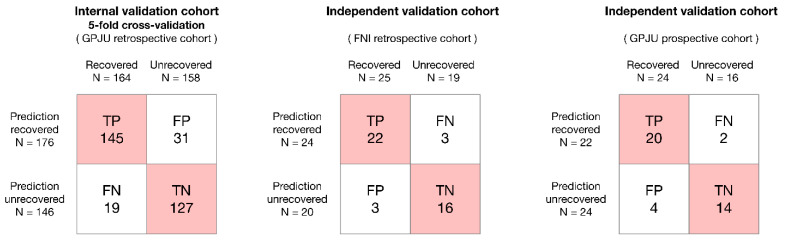
Confusion matrix in the training and validation cohorts.

We tested the model performance in two independent cohorts (Table 4). The cohorts include retrospectively collected data from FNI and prospectively collected data from GPJU. Patients in the FNI cohort had larger tumor and worse visual function than those in our training cohort. However, patients in the prospective GPJU cohort had smaller tumors and better visual function than those in our training cohort. The trained ensemble model yielded AUCs of 0.861 and 0.843 in the retrospective FNI and prospective GPJU validation cohorts, respectively. The corresponding accuracies, sensitivities, and specificities were 86.4%, 0.842, and 0.880 and 85.0%, 0.875, and 0.833 for the two validation cohorts, respectively (Table 3). The true-positive, true-negative, false-positive, and false-negative predictions in the training and independent validation cohorts are listed in Figure 3. Most cases can be correctly classified.

**Figure 4:**
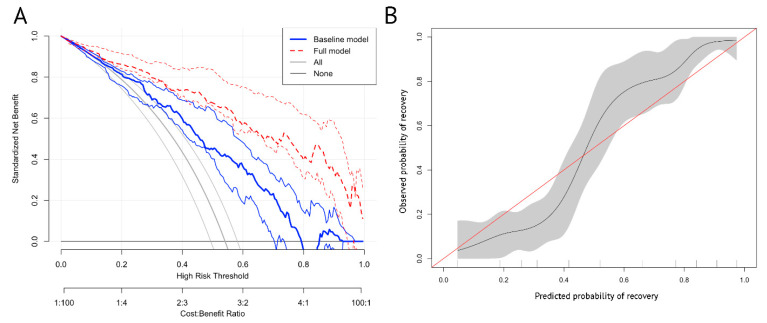
Decision support curve and calibration plot. (**A**) The curve presented that the net benefit of our full model was higher than the non-model or model only using the visual field as the predictor (baseline model). Standardized net benefit is a measure of utility that calculates a weighted sum of true positives and false positives, weighted according to the threshold. (**B**) The model showed good calibration with an intercept close to 0 and a slope close to 1. The width of the grey area represents the number of patients at each level of “predicted probability of recovery”.

We investigated the utility of our model by plotting a decision support curve. The curve presented that the net benefit of our full model was higher than the non-model or model only using the visual field as the predictor (baseline model). PREVOST provided greater net benefit than the competing extremes of intervening in all patients or none (Figure 4A). At most risk thresholds greater than 0.1, the full model provided significant improvement in net benefit compared with the baseline model. Moreover, the model showed good calibration with low Brier scores (0.055; Figure 4B).

**Figure 5:**
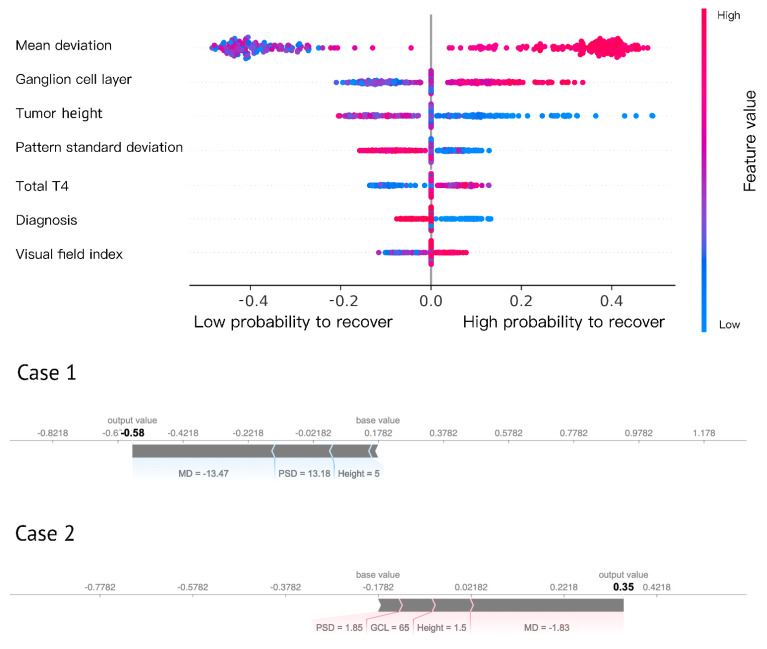
SHAP score-based model explanation. Every dot in the figure represents a patient. The X-axis represents the contribution to prediction (SHAP score). The variables were ordered by importance (width). Red (high) and blue (low) represent the values of the variables, e.g., for Ganglion cell layer, red means high and blue means low. Two representative cases: a severe visual field and pituitary macroadenoma contribute to the low probability of recovery (negative output) in Case 1, while a mild visual field defect, normal ganglion cell layer, and small tumor contribute to the high probability of recovery (positive output) in Case 2.

A model explanation using the SHAP score demonstrated that visual field, GCL, tumor height, total thyroxine, and diagnosis were the most important features in predicting visual outcome. We illustrate two cases in Figure 5, one recovered and the other unrecovered.

**Figure 6:**
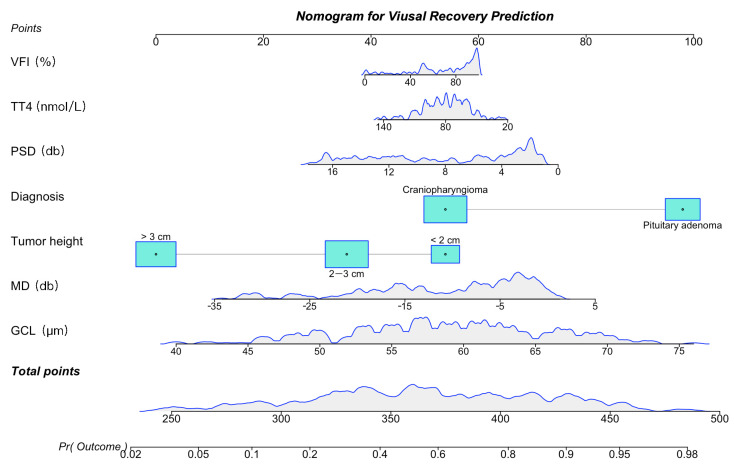
Nomogram for predicting visual outcome after transsphenoidal optic decompression. Physicians can add up corresponding scores using the graph and can obtain the recovery probability.

We simplified the model using these important features to construct a simple version during clinical usage. The AUC of the simple model was 0.874 [95%CI, 0.838–0.910], which was not significantly inferior to that of the original model. We constructed a nomogram based on the simple model (Figure 6). Physicians can add up corresponding scores using the graph and can obtain the recovery probability.

## 4. Discussion

We developed and independently validated PREVOST, which is, to our knowledge, the first risk-prediction algorithm specifically for visual outcomes in patients with sellar tumors. PREVOST can predict the risk of persistent visual deterioration from commonly recorded clinical information and available ophthalmic testing. The internal and external validations of PREVOST were good, with C statistics greater than 0.80. PREVOST displayed greater net benefit than alternative strategies across a range of feasible risk thresholds, although our results show that the full model should be used preferentially at most risk thresholds.

Previous studies have discussed various prognostic factors [9,10,11,12,13,14,15,16,17,18,19] about visual defects caused by compressive sellar region tumors. Age [5,14,25], duration of visual symptoms prior to surgery [9,12], whether the adenoma is secreting or non-secreting [25,26], tumor volume [10,27,28,29], pre-operative visual field deficit [9,15,19,25,27], retinal nerve fiber layer thickness [11,17,18,19,30], optic disc pallor [31,32,33], and functional MRI [13,16] were possible predictors discussed in one or several studies. However, these studies used small sample sizes, unquantified outcomes, or only a few possible predictors. In this study, however, the predictive model was developed by analyzing risk factors based on multiple factors.

Visual fields are among the most commonly included predictors in existing algorithms and are well-known contributors to visual risk, so we included them in PREVOST. Gnanalingham et al. [9] studied 41 patients with visual disturbance caused by pituitary adenomas and found that the extent of the visual recovery was mainly dependent on the preoperative visual field deficit. Yu et al. concluded that low preoperative mean deviation was one of the independent influencing factors for improving the visual field after pituitary adenomas resection [25]. Tuomas et al. also concluded that severe preoperative visual impairment resulted in poorer postoperative visual outcomes [27]. In accordance with past results, our study also established the prognostic value of preoperative visual fields. The duration of visual symptoms was another risk factor in previous studies [9,12], but it was not correlated with pre-operative visual function and was also excluded in the simplified model due to possible recall bias.

The prognostic value of GCL has been previously assessed by several researchers [11,17,18,19,30]. Maud Jacob et al. [11] evaluated 37 eyes of 19 patients suffering from pituitary adenomas and found that a lower RNFL thickness was a potent prognostic factor. The findings on RNFL thickness in our study were similar to the recently published research by Danesh-Meyer et al. [18], who studied 205 eyes from 107 patients and found that patients with normal preoperative RNFL thickness showed an increased propensity for visual recovery.

Tumor height was associated with visual recovery in several studies [10,27,28,29], and we included it in PREVOST. Blood-based predictors, such as cortisol and ACTH, were relatively infrequently included in visual risk-prediction algorithms. We found that the inclusion of blood-based predictors improved all predictive performance metrics. However, blood-based monitoring might not always be possible, and we found that the simple model still provided reliable performance estimates.

Patients and clinicians might prefer to tolerate a slightly higher risk threshold when the proposed intervention could be deemed more burdensome or might increase the risk of other adverse effects. The risk threshold for our PREVOST model was set to be 0.5. However, trials of treatments such as visual rehabilitation are scarce in these patients, but evidence suggests that such treatments might benefit visual outcomes [7,8].

The limitations of the study include non-universal representation and a lack of external prospective validation. We only included patients with craniopharyngiomas and pituitary adenomas in our study because these were the two major lesions that produce visual disturbance. Other cases, such as meningioma, could potentially be added to update the algorithm in future studies. Though the model was validated in an external cohort, with the two centers being similar in surgical volume and experience, the generalization of our model in other institutions is unknown. An external validation of PREVOST on prospective samples is required since simulation studies have suggested a minimum of 100 outcome events for an accurate validation analysis.

## 5. Conclusions

A new prognostic model for visual recovery after trans-sphenoidal sellar region tumor resection was developed based on an ensemble machine learning analytical approach. The score can become a valuable resource for healthcare professionals by identifying patients with a higher risk of persistent visual deficit. The large-scale and prospective application of the proposed model would strengthen its clinical utility and universal applicability in practice.

## Figures and Tables

**Table 1:** Overall characteristics of the cohort.

	Overall N = 159	Unrecovered Eyes N = 146	Recovered Eyes N = 172	*p*
Gender (male)	91 (57.2%)	93 (63.7%)	89 (51.7%)	0.103
Age (years old)	42.3 (16.2)	45.2 (16.5)	39.8 (15.4)	0.023
Body mass index (kg/m^2^)	24.1 (3.6)	24.3 (3.2)	24.2 (4.3)	0.850
Comorbidities				
Hypertension	12 (7.5%)	9 (6.2%)	15 (8.7%)	0.518
Diabetes Mellitus	7 (4.4%)	12 (8.2%)	2 (1.2%)	0.020
Disease duration (months)	8.0 [1.0, 100.0]	12.0 [1.0, 100.0]	6.0 [1.0, 72.0]	0.002
Tumor height (cm)	3.0 (1.0)	3.3 (1.0)	2.8 (0.9)	<0.001
Diagnosis				<0.001
Pituitary adenomas	63 (39.6%)	40 (27.4%)	86 (50.0%)	
Craniopharyngiomas	96 (60.4%)	126 (73.6%)	86 (50.0%)	
Laboratory test				
Hemoglobin (g/L)	129.4 (15.9)	128.2 (17.3)	130.4 (14.5)	0.349
Red Blood Cell (10^12^/L)	4.3 (0.5)	4.3 (0.5)	4.3 (0.5)	0.185
White Blood Cell (10^9^/L)	6.6 (2.1)	6.9 (2.2)	6.4 (2.1)	0.117
Sodium (mmol/L)	140.5 (4.7)	140.4 (4.7)	140.7 (4.7)	0.670
Albumin (g/L)	43.2 (5.15)	42.8 (5.9)	43.7 (4.4)	0.239
Creatinine (μmol/L)	68.1 (15.3)	68.9 (16.7)	67.4 (14.1)	0.386
ACTH (pg/mL)	25.1 [1.1, 197.8]	23.9 [1.1, 197.8]	28.1 [3.5, 92.5]	0.936
Cortisol (μg/dL)	7.6 [0.05, 21.4]	6.6 [0.05, 48.8]	8.4 [0.1, 104.6]	0.099
Prolactin (ng/mL)	24.7 [0.4, 470.0]	21.7 [0.5, 470.0]	26.6 [0.4, 470.0]	0.052
Free Thyroxine (pmol/L)	13.8 (4.5)	13.4 (4.8)	14.2 (4.2)	0.252
Total Thyroxine (nmol/L)	80.3 (22.1)	78.9 (23.8)	81.5 (20.6)	0.429
Ophthalmology				
Visual acuity	0.6 [0.1, 1.0]	0.6 [0.1, 1.0]	0.8 [0.1, 1.0]	0.784
Visual field				
Mean deviation (db)	−8.0 [−34.2, 1.3]	−14.6 [−34.2, −0.1]	−5.0 [−32.5, 1.3]	<0.001
Pattern standard deviation (db)	7.4 [1.1, 17.7]	11.2 [1.1, 17.7]	4.3 [1.1, 17.3]	<0.001
Visual field index	70.8 (28.3)	58.7 (29.6)	81.0 (22.5)	<0.001
Retinal Nerve Fiber Layer (μm)	96.2 (33.2)	91.9 (44.5)	99.8 (18.2)	0.163
Ganglion Cell Layer (μm)	58.7 (7.1)	56.6 (7.6)	60.5 (6.1)	<0.001

**Table 2:** Ophthalmic examinations in patients with different diagnoses and different eyes.

	Overall N = 159	Craniopharyngioma N = 96	Pituitary Adenoma N = 63	*p*
Visual acuity				
Left	0.6 [0.1, 1.0]	0.7 [0.1, 1.0]	0.2 [0.1, 1.0]	0.017
Right	0.6 [0.1, 1.0]	0.8 [0.1, 1.0]	0.5 [0.1, 1.0]	0.189
Visual field				
Left				
Mean Deviation (db)	−8.8 [−34.2, 1.1]	−9.1 [−32.5, 0.1]	−7.8 [−34.2, 1.1]	0.503
Pattern Standard Deviation (db)	7.4 [1.1, 17.3]	6.0 [1.2, 16.9]	9.1 [1.1, 17.3]	0.477
Visual Field Index	69.5 (29.0)	67.5 (31.2)	72.5 (25.3)	0.288
Right				
Mean Deviation (db)	−7.8 [−32.0, 1.3]	−8.6 [−32.0, 0.0]	−6.7 [−29.7, 1.3]	0.129
Pattern Standard Deviation (db)	7.5 [1.1, 17.7]	7.6 [1.1, 16.8]	6.5 [1.1, 17.7]	0.586
Visual Field Index	72.1 (27.6)	69.9 (28.8)	75.4 (25.6)	0.222
Ganglion cell layer (μm)				
Left	58.5 (7.0)	58.9 (7.5)	57.7 (6.3)	0.290
Right	58.9 (7.1)	58.9 (7.5)	59.1 (6.4)	0.874
Retinal nerve fiber layer (μm)				
Left	99.4 (33.2)	98.3 (40.9)	101.1 (15.6)	0.609
Right	93.0 (33.0)	96.1 (38.1)	88.2 (22.5)	0.139
Recovered eyes				
Left	84 (52.8%)	42 (43.8%)	42 (66.7%)	0.008
Right	88 (55.3%)	44 (45.8%)	44 (69.8%)	0.005

**Table 3:** Model performance using different algorithms.

	AUC	Accuracy	Sensitivity	Specificity
Training cohort (fivefold cross validation) GPJU retrospective cohort				
LASSO	0.854 [95% CI, 0.807–0.901]	0.777	0.759	0.792
Support Vector Machine	0.875 [95% CI, 0.824–0.927]	0.786	0.764	0.806
Linear Discriminant Analysis	0.846 [95% CI, 0.794–0.897]	0.774	0.761	0.784
Random Forest	0.901 [95% CI, 0.880–0.921]	0.837	0.809	0.861
Gradient Boosting	0.889 [95% CI, 0.862–0.901]	0.799	0.789	0.807
Neural Network	0.858 [95% CI, 0.816–0.900]	0.780	0.757	0.800
Ensemble Model	0.911 [95% CI, 0.885–0.938]	0.843	0.863	0.820
Independent cohort				
FNI retrospective cohort	0.861	0.864	0.842	0.880
GPJU prospective cohort	0.843	0.850	0.875	0.833

FNI: Fudan Neurosurgical Institute. GPJU: Gold Pituitary Joint Unit.

**Table 4:** Comparison among three cohorts.

	Retrospective GPJU N = 159	Retrospective FNI N = 22	Prospective GPJU N = 20
Gender (male)	91 (57.2%)	17 (%)	8 (51.7%)
Age (years old)	42.3 (16.2)	41.4 (16.5)	39.0 (14.5)
Tumor height (cm)	3.0 [1.0–6.0]	3.5 [1.0–5.5]	2.4 [1.0–5.8]
Diagnosis			
Pituitary adenomas	63 (39.6%)	22 (100.0%)	15 (75.0%)
Craniopharyngiomas	96 (60.4%)	0 (0.0%)	5 (25.0%)
Ophthalmology			
Visual acuity	0.6 [0.1, 1.0]	0.4 [0.1, 1.0]	0.6 [0.1, 1.0]
Visual field			
Mean deviation (db)	−8.0 [−34.2, 1.3]	−14.3 [−29.0, 0.0]	−5.4 [−30.7, 0.4]
Pattern standard deviation (db)	7.4 [1.1, 17.7]	12.0 [1.0, 18.8]	3.8 [1.4, 16.6]
Visual field index (%)	70.8 (28.3)	56.0 (27.0)	90.0 (27.0)
Retinal Nerve Fiber Layer (μm)	96.2 (33.2)	95.8 (16.3)	103.5 (53.0)
Ganglion Cell Layer (μm)	58.7 (7.1)	87.7 (10.3)	60.2 (8.5)
Outcome: recovered	54.1%	56.8%	60.0%

FNI: Fudan Neurosurgical Institute. GPJU: Gold Pituitary Joint Unit.

## Data Availability

De-identified data will be available upon request.

## Availability of Data and Material

De-identified data are available upon request.

## Code Availability

All statistical analyses were completed in R software version 3.4.2, and code is available upon request.
